# Validation of four candidate pancreatic cancer serological biomarkers that improve the performance of CA19.9

**DOI:** 10.1186/1471-2407-13-404

**Published:** 2013-09-03

**Authors:** Shalini Makawita, Apostolos Dimitromanolakis, Antoninus Soosaipillai, Ireena Soleas, Alison Chan, Steven Gallinger, Randy S Haun, Ivan M Blasutig, Eleftherios P Diamandis

**Affiliations:** 1 Department of Laboratory Medicine and Pathobiology, University of Toronto, Toronto, ON, Canada; 2 Department of Surgery, Mount Sinai Hospital, Toronto, ON, Canada; 3 Pathology and Laboratory Medicine, Mount Sinai Hospital, Toronto, ON, Canada; 4 Zane Cohen Familial Gastrointestinal Cancer Registry, Mount Sinai Hospital, Toronto, ON, Canada; 5 Department of Pharmaceutical Sciences, Winthrop P. Rockefeller Cancer Institute, University of Arkansas for Medical Sciences and Central Arkansas Veterans Healthcare System, Little Rock, AR, USA; 6 Department of Clinical Biochemistry, University Health Network, Toronto, ON, Canada; 7 Department of Pathology and Laboratory Medicine, Mount Sinai Hospital, 6th Floor, Room 6-201, Box 32, 60 Murray Street, Toronto, ON M5T 3L9, Canada

**Keywords:** Pancreatic cancer, Serum biomarkers, Biomarker validation, ELISA, Biomarker panel

## Abstract

**Background:**

The identification of new serum biomarkers with high sensitivity and specificity is an important priority in pancreatic cancer research. Through an extensive proteomics analysis of pancreatic cancer cell lines and pancreatic juice, we previously generated a list of candidate pancreatic cancer biomarkers. The present study details further validation of four of our previously identified candidates: regenerating islet-derived 1 beta (REG1B), syncollin (SYCN), anterior gradient homolog 2 protein (AGR2), and lysyl oxidase-like 2 (LOXL2).

**Methods:**

The candidate biomarkers were validated using enzyme-linked immunosorbent assays in two sample sets of serum/plasma comprising a total of 432 samples (Sample Set A: pancreatic ductal adenocarcinoma (PDAC, n = 100), healthy (n = 92); Sample Set B: PDAC (n = 82), benign (n = 41), disease-free (n = 47), other cancers (n = 70)). Biomarker performance in distinguishing PDAC from each control group was assessed individually in the two sample sets. Subsequently, multiparametric modeling was applied to assess the ability of all possible two and three marker panels in distinguishing PDAC from disease-free controls. The models were generated using sample set B, and then validated in Sample Set A.

**Results:**

Individually, all markers were significantly elevated in PDAC compared to healthy controls in at least one sample set (p ≤ 0.01). SYCN, REG1B and AGR2 were also significantly elevated in PDAC compared to benign controls (p ≤ 0.01), and AGR2 was significantly elevated in PDAC compared to other cancers (p < 0.01). CA19.9 was also assessed. Individually, CA19.9 showed the greatest area under the curve (AUC) in receiver operating characteristic (ROC) analysis when compared to the tested candidates; however when analyzed in combination, three panels (CA19.9 + REG1B (AUC of 0.88), CA19.9 + SYCN + REG1B (AUC of 0.87) and CA19.9 + AGR2 + REG1B (AUC of 0.87)) showed an AUC that was significantly greater (p < 0.05) than that of CA19.9 alone (AUC of 0.82). In a comparison of early-stage (Stage I-II) PDAC to disease free controls, the combination of SYCN + REG1B + CA19.9 showed the greatest AUC in both sample sets, (AUC of 0.87 and 0.92 in Sets A and B, respectively).

**Conclusions:**

Additional serum biomarkers, particularly SYCN and REG1B, when combined with CA19.9, show promise as improved diagnostic indicators of pancreatic cancer, which therefore warrants further validation.

## Background

Pancreatic cancer is the tenth most common cancer type and the fourth leading cause of cancer-related deaths [1]. Diagnosis of small, early-stage tumors that can be surgically resected offers patients the best chances for survival and can increase five-year survival rates from ~5% to 20-30%, or higher at specialized treatment centers [1,2]. Unfortunately, given the asymptomatic nature of its early stages, its aggressive disease course, and limitations of current detection technologies, fewer than 20% of patients are diagnosed with resectable disease.

Currently, detection of pancreatic cancer is based largely on various imaging modalities, such as computed tomography (CT), endoscopic ultrasound (EUS) and magnetic resonance imaging (MRI) [3,4]. More definitive preoperative diagnoses of pancreatic cancer typically requires invasive means such as endoscopic retrograde cholangiopancreatography (ERCP) which enables tissue sampling or endoscopic ultrasound-guided fine needle aspiration (EUS-FNA) [5,6]. The major drawback of all of these methods for the optimal management of pancreatic cancer patients is that they are primarily utilized after the onset of symptoms (i.e. predominantly after the onset of late-stage disease). They are also associated with relatively high operating costs, and can be somewhat time consuming and/or invasive in nature. In this regard, implementation of highly sensitive and specific biomarkers or marker panels for pancreatic cancer can further enhance detection strategies by offsetting many of the limitations described above [5,6].

The current clinically used marker for pancreatic cancer is carbohydrate antigen 19.9 (CA19.9). CA19.9 is a sialylated Lewis A-active pentasaccharide detected primarily on the surface of mucins in the serum of pancreatic cancer patients [7,8]. Although elevated CA19.9 levels have been associated with advanced stages of the disease, they have also been associated with benign and inflammatory diseases [8-10]. For early-stage pancreatic cancer detection, CA19.9 has a reported sensitivity of ~55% and is often undetectable in many asymptomatic individuals [7]. In addition, CA-19.9 is associated with Lewis antigen status and is absent in individuals with blood group Le(a^-^b^-^) (~10% of the general population) [7,11]. Taken together, CA19.9 alone lacks the necessary sensitivity and specificity for pancreatic cancer detection and according to the American Society of Clinical Oncology Tumor Markers Expert Panel, CA19.9 is recommended only for monitoring response to treatment in patients who had elevated levels prior to treatment [12].

With the aim of identifying new biomarkers for pancreatic cancer, we previously performed proteomic analysis of conditioned media (CM) from six pancreatic cancer cell lines, one ‘normal’ pancreatic ductal epithelial cell line and six pancreatic juice samples using two dimensional LC-MS/MS [13]. A total of 3479 nonredundant proteins were identified with high confidence. Three strategies were then utilized to mine the list of identified proteins for putative candidate pancreatic cancer biomarkers: (1) differential protein expression analysis between the cancer and normal cell lines using label-free protein quantification, (2) an integrative analysis, concentrating on the proteins consistently identified in the multiple pancreatic cancer biological fluids subjected to proteomics analysis, and (3) analysis of tissue specificity through mining of publically available databases [13]. Of the candidates identified in our previous work, the current study details the validation of four candidates, REG1B, SYCN, AGR2 and LOXL2. These four candidates were selected based on commercially available ELISA kits for validation, as well as preliminary verification studies in smaller sample sets as described in our previous publication for AGR2 [13], and conducted in-house thereafter for the other three candidates (data not shown).

## Methods

### Serum and plasma samples

This retrospective study population consisted of 432 individuals and comprised two sample sets, denoted A and B. Sample Set A consisted of 100 plasma samples from patients with established pancreatic ductal adenocarcinomas (PDAC or pancreatic cancer) and 92 samples from healthy controls that were non-blood relatives of pancreatic cancer patients). The samples were provided by Dr. Steven Gallinger’s group at the University of Toronto and collected at the Princess Margaret Hospital GI Clinic in Toronto, Canada, or from kits sent directly to consented patients recruited from the Ontario Pancreas Cancer Study at Mount Sinai Hospital following a standardized protocol. This protocol for sample collection was approved by the Institutional Review Boards of University Health Network and Mount Sinai Hospital. Blood was collected in ACD (anticoagulant) vacutainer tubes and plasma samples were processed within 24 hours of blood draw. To pellet the cells, blood samples were centrifuged at room temperature for 10 minutes at 913 X g. Immediately after centrifugation, the plasma samples were aliquoted into 250 uL cryotubes and stored in −80°C or liquid nitrogen until further use.

Sample Set B consisted of serum samples from the following groups: 82 PDAC patients, 41 patients with benign diseases (which included 10 patients with intraductal papillary mucinous neoplasms (IPMNs)), 10 total with adenomas of the pancreas (n = 8, mucinous/serous cystadenomas) and of tubulovillous adenoma of duodenum (n = 2), and 21 pancreatitis samples (primarily chronic)), 70 samples from patients with other malignancies (primarily GI malignancies such as colon, liver and stomach cancer) and 47 samples from non-cancer/disease-free controls as per self-reported questionnaires. Sample Set B was provided by Dr. Randy Haun at the Winthrop P. Rockefeller Cancer Institute, University of Arkansas for Medical Sciences. All samples were collected with informed consent and with approval from the respective Institutional Review Board of the University of Arkansas. A summary of sample characteristics is listed in Table 1.

**Table 1:** Sample characteristics

**Sample group**	**Source**	**Sample type**	**Sample characteristic**	**Total number of samples**	**Number of females/males**	**Median (Mean) Age**
**Sample Set A**	**Plasma**	**Healthy**	**n/a**^b^	**92**	**47/45**	**60.0 (60.7)**
		**PDAC**^**a**^	Early Stage (I & II)^c^	20	6/14	68.5 (67.1)
			**Total**	**100**	**37/63**	**65.5 (63.7)**
**Sample Set B**	**Serum**	**Disease-free**	**n/a**	**47**	**34/11**	**51.0 (50.2)**
		**PDAC**	Early Stage (I & II)^c^	40	22/18	68.0 (66.1)^d^
			**Total**	**82**	**44/38**	**63.5 (63.8)**
		**Benign**	Neoplasm/adenoma^e^	20	9/11	64.0 (60.0)
			Pancreatitis^f^	21	10/11	59.0 (56.8)
			**Total**	**41**	**19/22**	**62.0 (58.3)**
		**Other cancers**	Colon	33	14/19	62.0 (63.0)
			Liver	13	9/4	50.0 (56.2)
			Stomach	5	2/3	75.0 (71.8)
			Other^g^	19	7/12	68.0 (62.6)
			**Total**	**70**	**32/38**	**62.5 (62.3)**

^a^ PDAC, pancreatic ductal adenocarcinoma; ^b^ Not Applicable; ^c^ Stage was available for 47 PDAC samples from Sample Set A and 51 PDAC samples from Sample Set B; ^d^ One sample did not contain age information; ^e^ This group included intraductal papillary mucinous neoplasms (n = 10), serous/mucinous cystadenomas (n = 8), tubulovillous duodenal adenoma (n = 2); ^f^ Eighteen of 21 samples were listed as chronic pancreatitis; ^g^ Other includes ampullary cancer, Hodgkin’s lymphoma, renal cell carcinoma.

### Measurement of AGR2, REG1B, SYCN, LOXL2 and CA19.9 levels

Commercially available ELISA kits were purchased for AGR2, REG1B, SYCN and LOXL2 from USCN LifeSciences (AGR2: Catalogue # E2285Hu; SYCN: Catalogue E93879Hu; REG1B: Catalogue # E94674Hu; LOXL2: Catalogue # E95552Hu). ELISAs were performed according to manufacturer’s instructions with slight modifications. Briefly, 100 uL of sample was incubated in pre-coated 96-well plates for 2 hours at 37°C, along with standards. Samples were diluted in phosphate-buffered saline as instructed, using a 1:10 dilution for SYCN and AGR2, 1:100 dilution for LOXL2 and 1:2000 dilution for REG1B. Plates were washed twice using the wash buffer provided in the kits. A biotin-conjugated polyclonal secondary antibody specific for each of the proteins (detection reagent A from USCN kit) was prepared and incubated for 1 hour at 37°C. Following 4 washes, horseradish peroxidase (HRP) conjugated to avidin (detection reagent B from USCN kit) was prepared and incubated for 30 min at 37°C. The plates were washed 4 times and 90 uL of tetramethylbenzidine (TMB) substrate was added to each well. Wells were protected from light and incubated at 37°C for 10–15 min or until the two highest standards were not saturated (based on visual examination of color change). Fifty microliters of stop solution (sulphuric acid solution provided in USCN kit) was added and the color change was measured spectrophotometrically using a Perkin-Elmer Envision 2103 multilabel reader at a wavelength of 450 nm (540 nm measurements were used to determine background). CA19.9 levels were measured using a commercially available immunoassay (ELECSYS by Roche) and performed according to manufacturer’s instructions.

### Statistical analysis

All comparisons of medians between case and control groups were conducted using the Mann–Whitney-Wilcoxon test, as the distribution of concentrations deviated from normality. The Spearman’s rank correlation coefficient was used to determine association of markers with age in the healthy control group (n = 92) and Wilcoxon p-values were calculated to determine association of markers with gender in this group. The diagnostic value of the proteins was further assessed using receiver operating characteristic (ROC) curve analysis and area under the curve (AUC) calculations. Confidence intervals (95%) for AUC were calculated by using DeLong’s method for two correlated ROC curves. P-values comparing two AUCs were calculated by taking 2000 stratified bootstrap samples.

Multi-parametric models for combinations of markers were evaluated using a logistic regression model. The log_2_ transformed marker concentrations were used as predictors on a logistic regression model against the outcome (healthy vs PDAC). The estimated coefficients of the model were used to construct a composite score for each observation which was used for the construction of the ROC curves and subsequent analysis. PDAC versus non-cancer samples from Sample Set B was used as a training set from which models were derived and then validated in the PDAC versus healthy controls of Sample Set A. Models for early-stage PDAC compared to healthy controls were also assessed.

All parts of the statistical analysis were performed in the R environment (version 2.14.0) available from http://www.R-project.org. ROC curve analysis and comparisons between ROC curves was performed using the pROC package [14].

## Results

### Assay precision

Assay precision (reproducibility) was assessed through inclusion of four internal controls in each of the ELISA plates during the validation experiments (Additional file 1: Table S1). Coefficients of variation (CV) calculated for each of the four controls across the 7 plates utilized for each protein are shown in Additional file 1: Table S1. Overall, very good inter-assay reproducibility was shown for SYCN, AGR2, REG1B and LOXL2 assays with %CVs <20%, except for control 1 in AGR2 which had a %CV of 22% and control 2 in REG1B which had a %CV of 21%.

### Performance of SYCN, REG1B, AGR2, LOXL2 and CA19.9 analyzed individually in pancreatic cancer and control groups

All samples (n = 432) from the two sample sets described in the Methods section were subjected to ELISA analysis in parallel and on the same day for each candidate. Statistical analysis was conducted separately for Sample Sets A and B, as set A contained plasma samples, while set B contained serum samples, and they were collected/stored at different institutions. The following comparisons were made for Sample Set A: PDAC versus healthy controls (Table 2). The following comparisons were made for Sample Set B: PDAC versus non-cancer/disease free controls (Table 2), PDAC versus benign disease (Additional file 1: Table S2), and PDAC versus other cancers (Additional file 1: Table S2). Below is a summary of results by candidate tested from all comparisons made.

**Table 2:** Significance tests and AUC values for AGR2, SYCN, REG1B, LOXL2 and CA19.9 analyzed in PDAC versus healthy controls of Sample Set A and B

**Comparison group**	**Marker**	**Sample set**	**Median healthy**	**Median PDAC**	**Median Ratio**	**Wilcoxon**	**AUC**^b^	**Lower 95% CI**	**Upper 95% CI**
						**p-value **^a^			
**PDAC versus healthy controls**	**SYCN**	A	3.82	8.22	2.15	8.38E-07	0.71	0.63	0.78
		B	3.56	12.72	3.57	5.94E-08	0.79^c^	0.70	0.87
	**AGR2**	A	184.85	179.55	0.97	0.38	0.46	0.38	0.54
		B	117.40	173.90	1.48	0.00129	0.67	0.58	0.76
	**REG1B**	A	4364.00	15232.00	3.49	1.20E-08	0.74	0.67	0.80
		B	4582.00	25380.00	5.54	4.52E-08	0.79^c^	0.70	0.86
	**LOXL2**	A	139.45	172.25	1.24	0.019	0.60	0.52	0.68
		B	106.8	110.70	1.04	0.449	0.54	0.44	0.64
	**CA19.9**	A	6.00	59.05	9.84	9.54E-16	0.82	0.76	0.88
		B	14.75	144.625	9.81	3.46E-10	0.83^c^	0.76	0.90

^a^ The p-value refers to a comparison between PDAC and Healthy subgroups (Mann–Whitney non-parametric test). Sample sizes are provided in Table 1. Confidence intervals for AUC were calculated by taking 2000 stratified bootstrap samples; ^b^ AUC, area under the receiver operating characteristic curve; PDAC, pancreatic ductal adenocarcinoma (analogous to use of the term pancreatic cancer elsewhere in this report); ^c^ No significant difference in AUC was noted between the AUCs of SYCN and REG1B with that of CA19.9 (p > 0.4).

SYCN was significantly increased in PDAC when compared to healthy controls/disease-free samples of both sample sets (p = 8.38E-07 and p = 5.94E-08 for Sample Sets A and B, respectively) (Table 2). SYCN was also significantly increased in PDAC compared to the benign disease group (p = 0.014, Additional file 1: Table S2). No significant difference was found between PDAC and the other cancer group (Additional file 1: Table S2). SYCN performed best to discriminate PDAC from healthy/disease-free controls, with an area under the curve (AUC) of 0.79 (95% confidence intervals (CI) of 0.70-0.87) in Sample Set B.

REG1B performed similarly to SYCN in the tested samples. REG1B was also significantly elevated in the comparisons between PDAC and healthy/disease-free controls of both sample sets (p = 1.20E-08 and p = 4.52E-08 in Sample Sets A and B, respectively) (Table 2), as well as the comparison between PDAC and benign disease (p = 0.0085, Additional file 1: Table S2). Like SYCN, REG1B also showed no significant difference in PDAC versus other cancers (Additional file 1: Table S2). REG1B also performed best in discriminating PDAC from healthy/disease-free samples with an AUC of 0.79 (95% CI 0.70-0.86) in Sample Set B.

AGR2 was significantly increased in PDAC compared to healthy/disease-free controls in one of the two sample sets (p = 0.00129 in Sample Set B, Table 2). AGR2 was also significantly increased in PDAC compared to the benign disease group and PDAC compared to the other cancer group (p = 2.11E-06 and p = 4.54E-10, respectively, Additional file 1: Table S2). Interestingly, amongst all comparisons, AGR2 performed best in PDAC versus other cancers with an AUC of 0.79 (95% CI 0.72-0.86), followed by PDAC versus benign disease (AUC of 0.76).

LOXL2 was significantly elevated in PDAC versus healthy controls of Sample Set A (p = 0.019, Table 2); however this marker showed no significant difference in the comparisons between the other groups. Levels of CA19.9 were also assessed in the 432 samples for comparison purposes. Overall, individually, CA19.9 had the greatest AUC in comparison to the other tested markers for each comparison in both Sample Sets, with an AUC of 0.82 and 0.83 in the PDAC versus healthy/disease-free controls (Table 2), AUC of 0.87 in the PDAC versus benign disease group and 0.81 in the PDAC versus other cancer group (Additional file 1: Table S2). No significant difference in AUCs was found between SYCN, REG1B and CA19.9 in discriminating PDAC from disease-free controls (p ≥ 0.4) of Sample Set B (Table 2), and between AGR2 and CA19.9 (p = 0.69) in discriminating PDAC from other cancers (Additional file 1: Table S2).

Since Sample Set A contained plasma samples and Set B contained serum samples, they were analyzed separately; however upon performing a combined analysis for verification purposes of the healthy (n = 139) and PDAC (n = 132) samples from Sample Sets A and B, a similar trend was seen, with CA19.9, SYCN and REG1B showing significant differences between healthy and PDAC (CA19.9, p = 1.12E-24, AUC of 0.83; SYCN, p = 8.91E-14, AUC of 0.74; REG1B, p = 5.51E-16, AUC of 0.76).

### Association of biomarkers with age and gender

To determine if age had an effect on marker levels, the Spearman’s rank correlation coefficient was used to examine the correlation of marker concentrations with age in the healthy control group (sample set A, n = 92). The marker levels of none of the candidates (SYCN, AGR2, REG1B, or LOXL2) showed a significant correlation with age (Additional file 1: Table S3). CA19.9 levels were also not correlated with age in the studied samples. Additionally, no significant difference was noted in marker levels between males and females in this group (Additional file 1: Table S3).

### Biomarker panel modeling

**Figure 1:**
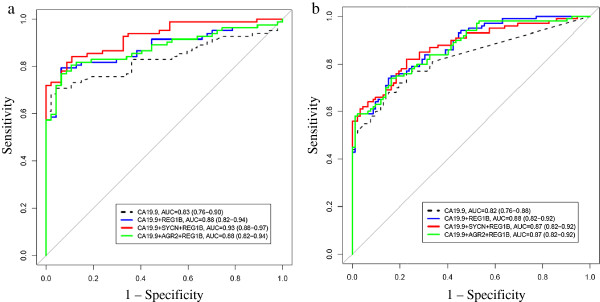
**Biomarker modeling in training (Sample Set B) and validation (Sample Set A) sets.** Biomarker models were generated for all two and three marker combinations in Sample Set B (Table 3). These models were then validated in PDAC versus healthy/non-cancer group of Sample Set A (Table 4). Displayed are three models which showed a significant improvement in AUC to that of CA19.9 alone in both training **(1a)** and validation sets **(1b)**. Confidence intervals (CI) for AUC were calculated using DeLong’s method. P-values are given in Table 4.

Multi-parametric models for combinations of markers were evaluated using log_2_ transformed marker concentrations as predictors on a logistic regression model against the outcome (healthy vs PDAC). Biomarker panels with and without CA19.9 were constructed using the non-cancer (n = 47) versus PDAC (n = 82) groups of Sample Set B as a training set since sample size of the comparison groups were smaller, and then applied to the healthy (n = 92) and PDAC (n = 100) groups of Sample Set A for validation. Models for all two and three marker panels (twenty models in total) from the training set are listed in Table 3. Ten models resulted in an AUC that was greater than that of CA19.9 alone. All models were validated in Sample Set A, resulting in three combinations, REG1B + CA19.9, SYCN + REG1B + CA19.9, and AGR2 + REG1B + CA19.9, which were found to significantly improve the AUC of CA19.9 alone (p = 0.001, p = 0.030, p = 0.004, respectively) (Table 4). Figures 1a and b show the ROC curves of these three models in the training and validation sets, respectively. The models were also applied to PDAC versus benign and PDAC versus other GI cancer groups (Additional file 1: Tables S4 and S5); however they did not improve the accuracy in these other comparisons.

**Table 3:** Biomarker modeling in training set (Sample Set B)

**Biomarker combination**^a^	**AUC**^b^**of combination**	**Lower 95% confidence interval**	**Upper 95% confidence interval**	**Specificity at 9% sensitivity**	**Sensitivity at 95% specificity**
CA19.9 + SYCN + REG1B	0.926	0.880	0.965	0.509	0.739
CA19.9 + SYCN + AGR2	0.919	0.869	0.959	0.515	0.771
CA19.9 + SYCN	0.918	0.864	0.958	0.502	0.771
CA19.9 + SYCN + LOXL2	0.918	0.871	0.959	0.479	0.779
CA19.9 + REG1B + LOXL2	0.879	0.814	0.936	0.219	0.727
CA19.9 + REG1B	0.878	0.815	0.933	0.261	0.689
CA19.9 + AGR2 + REG1B	0.877	0.816	0.932	0.268	0.688
CA19.9 + LOXL2	0.844	0.773	0.908	0.065	0.744
CA19.9 + AGR2 + LOXL2	0.844	0.772	0.910	0.065	0.744
CA19.9 + AGR2	0.835	0.762	0.902	0.046	0.707
CA19.9	0.833	0.757	0.902	0.048	0.699
SYCN + REG1B + LOXL2	0.826	0.747	0.896	0.250	0.395
SYCN + REG1B	0.823	0.745	0.893	0.248	0.356
SYCN + AGR2 + REG1B	0.819	0.741	0.891	0.261	0.359
SYCN + AGR2 + LOXL2	0.800	0.716	0.875	0.149	0.306
SYCN + AGR2	0.794	0.712	0.874	0.159	0.276
SYCN + LOXL2	0.793	0.712	0.870	0.179	0.282
REG1B	0.790	0.705	0.862	0.300	0.326
SYCN	0.790	0.703	0.868	0.201	0.220
AGR2 + REG1B	0.781	0.697	0.859	0.289	0.312
REG1B + LOXL2	0.779	0.694	0.854	0.186	0.369
AGR2 + REG1B + LOXL2	0.779	0.694	0.853	0.256	0.345
AGR2 + LOXL2	0.675	0.589	0.764	0.106	0.175
AGR2	0.671	0.576	0.763	0.087	0.130
LOXL2	0.540	0.439	0.644	0.033	0.218

^a^ Biomarker models were generated for each of the above combinations for the PDAC (n = 82) versus the disease-free (n = 47) groups of Sample Set B and ordered from greatest to lowest AUC. Confidence intervals (CI) for AUC were calculated using DeLong’s method. The models from the combinations of two or three markers were then validated in the PDAC versus healthy groups of Sample Set A (Table 4); ^b^ AUC, area under the receiver operating characteristic curve; PDAC, pancreatic ductal adenocarcinoma.

**Table 4:** Biomarker modeling in independent validation set (Sample Set A)

**Biomarker combination**^a^	**AUC**^b^**of combination**	**Lower 95% confidence interval**	**Upper 95% confidence interval**	**p-value of AUC of panel compared to AUC of CA19.9**
**CA19.9 REG1B**	**0.875**	**0.825**	**0.918**	**0.001**
**CA19.9 SYCN REG1B**	**0.873**	**0.823**	**0.918**	**0.033**
**CA19.9 AGR2 REG1B**	**0.869**	**0.816**	**0.913**	**0.004**
CA19.9 REG1B LOXL2	0.859	0.803	0.907	0.071
CA19.9 SYCN AGR2	0.858	0.804	0.907	0.117
CA19.9 SYCN	0.857	0.804	0.905	0.157
CA19.9 SYCN LOXL2	0.850	0.792	0.901	0.276
CA19.9 AGR2	0.824	0.764	0.883	0.946
CA19.9	0.824	0.765	0.877	1.000
CA19.9 AGR2 LOXL2	0.805	0.741	0.863	0.296
CA19.9 LOXL2	0.803	0.740	0.864	0.246
SYCN REG1B	0.782	0.716	0.845	0.297
SYCN REG1B LOXL2	0.776	0.707	0.842	0.264
SYCN AGR2 REG1B	0.774	0.708	0.834	0.243
REG1B LOXL2	0.747	0.677	0.813	0.086
AGR2 REG1B LOXL2	0.709	0.636	0.779	0.009
SYCN AGR2	0.706	0.634	0.778	0.009
SYCN AGR2 LOXL2	0.702	0.622	0.771	0.008
SYCN LOXL2	0.701	0.625	0.775	0.011
AGR2 REG1B	0.680	0.600	0.757	0.002
AGR2 LOXL2	0.582	0.493	0.660	0.000

^a^Biomarker models for two and three marker combinations generated in PDAC versus disease-free controls of Sample Set B and presented in Table 3 were validated in PDAC (n = 100) versus healthy controls (n = 92) of Sample Set A and ordered from greatest to lowest AUC. Confidence intervals (CI) for AUC were calculated using DeLong’s method. The top three models showed a significant improvement in AUC to that of CA19.9 alone. P-values were calculated by taking 2000 stratified bootstrap samples; ^b^AUC, area under the receiver operating characteristic curve; PDAC, pancreatic ductal adenocarcinoma.

### Levels of candidates in PDAC with CA19.9 values within normal range

CA19.9 is not expressed in approximately 10% of the general population that are Lewis antigen negative [7,11]. As a result, it is not elevated in all PDAC cases. Additionally, some patients that are Lewis antigen positive do not have elevated CA19.9. In this regard, we examined levels of our tested markers specifically in PDAC cases that had CA19.9 within the normal range (i.e. <37 Units/ mL) (Additional file 1: Table S6). Of the total 182 PDAC cases from both sample sets, 69 cases (38%) had CA19.9 levels that were within the normal range (<37 Units/mL; n = 45 PDAC cases in Sample Set A and n = 24 PDAC cases in Sample Set B). In this group, SYCN and REG1B were significantly increased in a proportion of patients with PDAC, with SYCN showing the greatest ability to capture cases missed by CA19.9 with an AUC of 0.67 and 0.84 in the Sample Sets A and B, respectively. At a cutoff of 13.96 ug/L and 17.4 ug/L, SYCN had a specificity of 90% in sample sets A and B, respectively, and was able to capture approximately one third of PDAC cases missed by CA19.9 (Additional file 1: Table S6).

### Distribution of candidates in early-stage PDAC

Of the total 182 PDAC samples used in the study, 98 contained clinical information pertaining to stage and 60 were listed as as stage I and II (early-stage pancreatic cancer according to the American Joint Committee on Cancer Staging [15]; n = 20 in Sample Set A and n = 40 in Sample Set B). In these samples, CA19.9 and SYCN performed comparably in discriminating PDAC from healthy/disease-free controls (AUC_SYCN_ = 0.73 and AUC_CA19.9_ = 0.76 (p = 0.81) in Sample Set A and AUC_SYCN_ = 0.81 and AUC_CA19.9_ = 0.80 (p = 0.96) in Sample Set B (Additional file 1: Tables S7 and S8)). The combination of SYCN + REG1B + CA19.9 showed the greatest AUC in both sample sets, (AUC of 0.87 and 0.92 in Sets A and B, respectively) and the following combinations performed best with sensitivities of 72-73% in Sample Set B at a specificity of 95%: CA19.9 + SYCN, CA19.9 + SYCN + AGR2 and CA19.9 + SYCN + LOXL2 (Additional file 1: Tables S7 and S8). Stage information for a large number of samples was unknown, therefore comparison between early and late stage was not performed.

## Discussion

Due to the lack of a single highly sensitive and specific marker for many diseases, including for various measurable outcomes of pancreatic cancer, research has shifted to the development of panels of markers to achieve improved performance [16]. In the current study, four pancreatic cancer biomarker candidates (SYCN, REG1B, AGR2 and LOXL2) delineated through our previous integrated proteomics analysis of cell line conditioned media and pancreatic juice [13], were validated in two sample sets of serum/plasma containing a total of 432 samples. Individually, CA19.9 performed best when compared to the tested candidates; however in combination, three panels were found to significantly improve the performance of CA19.9 in discriminating healthy from PDAC in a training and validation set (Table 4, Figure 1). Additionally, several of the analyzed candidates, particularly SYCN and REG1B show the ability to capture PDAC cases missed by CA19.9 (Additional file 1: Table S6). Several panels were also identified which significantly improved the diagnostic accuracy of CA19.9 for discriminating early-stage from disease-free subjects (Additional file 1: Tables S7 and S8). In terms of distinguishing PDAC from benign disease and PDAC from other cancers, aside from CA19.9, AGR2 showed the best discrimination between these two groups (Additional file 1: Table S2).

**Figure 2:**
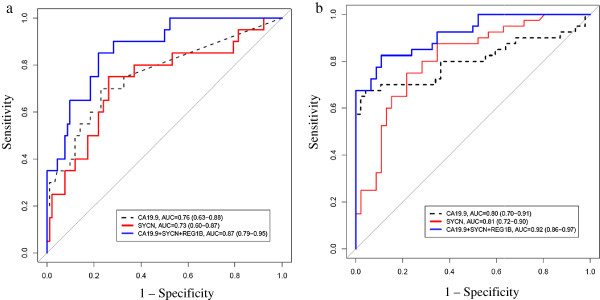
**Performance of SYCN, CA19.9 and the panel of SYCN + REG1B + CA19.9 in early stage (I/II) versus healthy/disease-free.** Biomarker performance was assessed in clinically confirmed early-stage (I/II) PDAC samples compared to healthy controls/disease-free individuals in Sample Set A (n = 20 PDAC and n = 92 healthy) **(a)** and Sample Set B (n = 40 PDAC and n = 47 disease-free) **(b)**. Displayed are the ROC curves for CA19.9 and SYCN, which performed comparably in the two sample sets (p = 0.81 and p = 0.96 showing no significant difference in AUCs of the two curves in Sample Set A and B, respectively). Also displayed is the ROC curve for the panel SYCN + REG1B + CA19.9, which showed the greatest AUC of all two and three marker combinations in both sample sets. Confidence intervals (CI) for AUC were calculated using DeLong’s method. Sensitivity and specificity are given in Additional file 1: Tables S7 and S8.

Syncollin is a protein that is highly expressed in pancreatic acinar granules. Specifically, it is a zymogen granule protein found on the inner surface of the granule membrane with a role in the concentration and maturation of zymogens, as well as in the regulation of exocytosis [17,18]. A few recent studies have described its presence and function in granules in other tissue types [19]. Syncollin has been identified in a qualitative proteomic analysis of pancreatic juice from patients with pancreatic cancer, and was elevated in serum from a murine model of pancreatic cancer [20,21]; however to our knowledge, this is the first report of its study and extended validation through ELISAs in human serum. In the present study SYCN was significantly elevated in patients with pancreatic cancer when compared to healthy controls in both sample sets, as well as when compared to benign disease controls. SYCN also showed the ability to best capture samples which had CA19.9 within normal limits (Figure 2), and was significantly elevated in early PDAC when compared to healthy controls. Given that the majority of pancreatic cancers are believed to arise from ductal cells (or possibly acinar cells that undergo acinar-to-ductal metaplasia) [22,23], elevation of SYCN in the circulation may be a secondary effect of the growing tumor through local tissue destruction. In pancreatic cancer, this has been recently studied for the protein transthyretin (TTR), an islet cell protein that is elevated in pancreatic juice from pancreatic cancer patients through destruction of islet cell architecture in the presence of invasive cancer [24]. A similar mechanism of local tissue destruction causing increased release of the protein may be occurring with SYCN. However, the unavailability of stage information for many of the PDAC cases prevented a comparison of early versus late stage SYCN levels in the present study and therefore, firm conclusions cannot be made regarding this at the present time. REG1B belongs to a family of proteins, encoded by the human *REG* genes, that is present in pancreatic acinar cells and promotes regeneration of pancreatic islets [25]. REG family members have been associated with pancreatic cancer or related diseases in the past. REG1A, a protein that is highly similar to REG1B has been implicated in pancreatitis, and other REG family members such as REGIV and hepatocarcinoma-intestine-pancreas/pancreatitis associated protein I (HIP/PAPI) have shown potential diagnostic utility for pancreatic cancer [26,27]. REGIV and REG1A have also been shown to be elevated in gastric cancer and may serve as prognostic indicators [28-30]. Both REG1A and REGIII were also found elevated in plasma from a mouse model of pancreatic cancer [21]. To the best of our knowledge, REG1B expression in serum has not been studied as a pancreatic cancer biomarker. In the present study, REG1B was significantly elevated in pancreatic cancer serum/plasma compared to healthy and benign disease controls, and it was a component of all three panels found to significantly improve the AUC of CA19.9 in our training and validation analyses (Tables 3 and 4).

Both REG1B and SYCN were identified in our previous proteomics discovery work as candidate pancreatic cancer biomarkers due to their presence in pancreatic juice from PDAC patients along with their identification as proteins highly tissue specific to the pancreas based on mining of several tissue specificity databases [13]. Interestingly, both proteins performed similarly in the validation study presented in this paper, being significantly elevated in comparisons between PDAC and healthy and PDAC and benign controls; however neither protein was able to significantly differentiate other cancers from PDAC in the studied samples. The ‘other cancer’ group in this study had a large fraction of serum samples from colon cancer patients. SYCN has not previously been studied in other cancers; however REG family gene expression, although not REG1B specifically, has been shown previously to be elevated in colon cancer and inflammatory bowel disease [31,32].

AGR2 is a protein initially identified in *Xenopus laevis* that was shown to be crucial during ectoderm developmental stages of embryogenesis for formation of anterior structures [33]. AGR2 is a member of the protein disulfide isomerase (PDI) family, found localized to both the endoplasmic reticulum and cell surface [33]. Its role in normal human structures is still somewhat unclear; however it has been implicated in many cancer types, including pancreatic cancer [34-36]. In pancreatic cancer cells, AGR2 has been shown to be involved in invasion and dissemination through posttranscriptional regulation of cathepsins D and B [34]. Recently, Chen et al. [37] found AGR2 to be overexpressed in pancreatic juice from patients with pancreatic intraepithelial neoplasia – III (PanIN3) and their ELISA results showed this protein to have potential diagnostic utility for pancreatic cancer in pancreatic juice; however these findings did not translate into their serum analysis. AGR2 was highly overexpressed in our previous integrated proteomic analysis of cell lines and pancreatic juice as well, and our preliminary verification studies showed it to be significantly elevated in plasma from pancreatic cancer patients [13]. In the present study, although significantly elevated in one sample set, AGR2 performed somewhat poorly in discriminating PDAC from healthy/non-cancer controls; however interestingly, it performed best, after CA19.9, in discriminating benign and other cancers from PDAC (Additional file 1: Table S2). AGR2 is a protein shown to promote tumor growth, cell transformation and migration [38]; however it is unclear as to why AGR2 was able to distinguish other cancers from PDAC in this study. As mentioned above, a large number of samples in the ‘other cancer’ group were colon cancer samples and it is possible that AGR2 serum levels are increased at higher levels in PDAC compared to colon cancer. A study from 2006 [39], also shows AGR2 mRNA levels to be downregulated in colon cancer; however firm conclusions cannot be made.

LOXL2 is an extracellular matrix protein involved in the epithelial to mesenchymal transition of cells and is highly expressed in desmoplastic/fibrotic stroma [40]. It has been shown to be upregulated in many cancer types and is believed to play a role in cancer metastasis. LOXL2 silencing in pancreatic cancer cells has shown improved sensitivity to gemcitabine therapy [41] and decreased tumor growth in gastric cancer [42]. In our serum/plasma analysis, the general ability of LOXL2 to distinguish PDAC from controls was not significant.

Validation of biomarkers and translation of markers from the bench to the clinic is a rigorous process [43]. The goal of this study was to preliminarily validate a set of the candidates identified in our previous proteomics work [13]. Of the candidates validated, several were able to significantly distinguish between case and control groups, with multiple panels demonstrating the ability to significantly improve the performance of CA19.9.

## Conclusions

According to recent research, there is at least a ten-year window during early-stage pancreatic cancer development, followed by another seven years before it metastasizes [44]. The greatest applicability of markers for the early detection of pancreatic cancer would likely be as a pretest to an imaging modality in screening and surveillance programs for detecting developing pancreatic cancers in high-risk patient groups. One of the limitations of this study was the lack of staging information for all cases. In this regard, further validation of the candidates and panels presented in this study is warranted in larger sample sets of individuals with early-stage disease, as well as those with precursor lesions such as PanIN lesions. Additionally, consideration of the markers/panels presented in this study for other measurable outcomes of pancreatic cancer, such as monitoring response to treatment and assessing disease recurrence is also warranted.

## Abbreviations

REG1B: Regenerating islet-derived 1 beta; SYCN: Syncollin; AGR2: Anterior gradient homolog 2 protein; LOXL2: Lysyl oxidase-like 2; ELISA: Enzyme-linked immunosorbent assays; PDAC: Pancreatic ductal adenocarcinoma; CA19.9: Carbohydrate antigen 19.9; AUC: Area under the curve; CI: Confidence interval; PanIN: Pancreatic intraepithelial neoplasm; CT: Computed tomography; EUS: Endoscopic ultrasound; MRI: Magnetic resonance imaging; ERCP: Endoscopic retrograde cholangiopancreatography; FNA: Fine needle aspiration; FAMMM: Familial atypical multiple mole melanoma; CM: Conditioned media; LC-MS/MS: Liquid chromatography tandem mass spectrometry; PIGR: Polymeric immunoglobulin receptor; GI: Gastrointestinal; IPMN: Intraductal papillary mucinous neoplasms; SEC: Size exclusion chromatography; HRP: Horseradish peroxidase; TMB: Tetramethylbenzidine; TTR: Transthyretin.

## Competing interests

The authors declare that they have no competing interests.

## Authors’ contributions

SM participated in the study design, performed experiments, analyzed data and drafted the manuscript. AD participated in the study design, performed statistical analyses and assisted with manuscript preparation. AS performed experiments and assisted with data analysis. IS assisted with experiments and data acquisition. AC assisted with experiments and data acquisition. SG provided plasma samples and participated in critical revision of manuscript. RSH provided serum samples and participated in critical revision of manuscript. IMB participated in the study design and manuscript revision. EPD supervised the project, participated in the study design, interpretation of results and revision/final review of manuscript. All authors read and approved the final manuscript.

## Pre-publication history

The pre-publication history for this paper can be accessed here:

http://www.biomedcentral.com/1471-2407/13/404/prepub

## Supplementary Material

Additional file 1: Table S1 Concentration, mean, standard deviation and %CVs of internal controls for each protein - assessment of inter-assay reproducibility. **Table S2** Sample characteristics, significance tests and AUC values for AGR2, SYCN, REG1B, LOXL2 and CA19.9 analyzed in Sample Set B for comparisons of PDAC versus benign and PDAC versus other cancers. **Table S3** Association of biomarkers with age and gender. **Table S4** Biomarker modeling in PDAC versus benign disease. **Table S5** Biomarker modeling in PDAC versus other cancers. **Table S6** Assessment of marker performance in 69 PDAC samples with CA19.9 levels within normal limits (<37 Units/mL). **Table S7** Marker performance in Early Stage (I/II) versus Healthy of Sample Set A. **Table S8** Marker performance in Early Stage (I/II) versus Disease-free of Sample Set B. Click here for file
