# Supplementary material for: Validation of four candidate pancreatic cancer serological biomarkers that improve the performance of CA19.9
Source: BMC Cancer. 2013 Sep 3;13:404. doi: 10.1186/1471-2407-13-404 (PMC3847832; doi:10.1186/1471-2407-13-404)
Supplement: Additional file 1: Table S1 — Concentration, mean, standard deviation and %CVs of internal controls for each protein - assessment of inter-assay reproducibility. Table S2 Sample characteristics, significance tests and AUC values for AGR2, SYCN, REG1B, LOXL2 and CA19.9 analyzed in Sample Set B for comparisons of PDAC versus benign and PDAC versus other cancers. Table S3 Association of biomarkers with age and gender. Table S4 Biomarker modeling in PDAC versus benign disease. Table S5 Biomarker modeling in PDAC versus other cancers. Table S6 Assessment of marker performance in 69 PDAC samples with CA19.9 levels within normal limits (<37 Units/mL). Table S7 Marker performance in Early Stage (I/II) versus Healthy of Sample Set A. Table S8 Marker performance in Early Stage (I/II) versus Disease-free of Sample Set B. [file 1471-2407-13-404-S1.doc]

**Supplemental Table 1. Concentration, mean, standard deviation and %CVs of internal controls for each protein - assessment of inter-assay reproducibility**

|  | | | **SYCN** | | | | | | | | **AGR2** | | | | | | | | **REG1B** | | | | **LOXL2** | | | |
| --- | --- | --- | --- | --- | --- | --- | --- | --- | --- | --- | --- | --- | --- | --- | --- | --- | --- | --- | --- | --- | --- | --- | --- | --- | --- | --- |
|  |  |  | **CONTROL NUMBER** | | | | | | | | **CONTROL NUMBER** | | | | | | | | **CONTROL NUMBER** | | | | **CONTROL NUMBER** | | | |
|  |  |  | **1^a^** | | **2^a^** | | **3^a^** | | **4^a^** | | **1^a^** | | **2^a^** | | **3^a^** | | **4^a^** | | **1^a^** | **2^a^** | **3^a^** | **4^a^** | **1^a^** | **2^a^** | **3^a^** | **4^a^** |
| **ELISA PLATE NUMBER** | **1** | | 2.10 | | 1.12 | | 1.94 | | 1.05 | | 29.29 | | 21.54 | | 42.50 | | 34.98 | | 4.49 | 1.15 | 19.87 | 2.47 | 16.99 | 6.70 | 4.58 | 2.61 |
|  |  |  | 1.92 | | 1.07 | | 1.90 | | 1.02 | | 27.81 | | 17.22 | | 42.04 | | 36.37 | | 4.39 | 0.80 | 17.82 | 2.82 | 14.83 | 5.69 | 4.46 | 2.71 |
|  | **2** | | 2.17 | | 1.15 | | 2.13 | | 1.03 | | 33.35 | | 24.22 | | 47.06 | | 31.69 | | 3.97 | 0.68 | 14.37 | 1.95 | 14.18 | 5.90 | 4.90 | 2.22 |
|  |  |  | 2.08 | | 0.94 | | 1.94 | | 1.08 | | 28.61 | | 20.67 | | 49.27 | | 30.95 | | 3.94 | 0.56 | 12.99 | 1.99 | 15.97 | 6.63 | 4.30 | 2.51 |
|  | **3** | | 2.21 | | 1.21 | | 1.82 | | 1.13 | | 25.88 | | 21.18 | | 39.39 | | 28.73 | | 3.44 | 0.64 | 12.59 | 2.31 | 15.07 | 4.91 | 3.92 | 2.88 |
|  |  |  | 2.19 | | 1.09 | | 1.91 | | 1.16 | | 24.06 | | 15.63 | | 40.22 | | 30.97 | | 3.36 | 0.74 | 11.68 | 2.32 | 12.53 | 5.42 | 3.77 | 2.33 |
|  | **4** | | 2.14 | | 1.17 | | 2.09 | | 1.09 | | 23.13 | | 17.08 | | 34.29 | | 28.99 | | 2.97 | 0.63 | 15.68 | 2.09 | 15.09 | 5.75 | 3.67 | 2.16 |
|  |  |  | 2.21 | | 1.04 | | 1.95 | | 1.16 | | 18.37 | | 13.18 | | 34.27 | | 29.40 | | 2.73 | 0.55 | 13.86 | 2.12 | 14.98 | 5.18 | 3.35 | 2.01 |
|  | **5** | | 1.98 | | 1.10 | | 2.08 | | 1.19 | | 15.81 | | 16.31 | | 29.42 | | 20.96 | | 3.61 | 0.93 | 19.95 | 2.87 | 11.35 | 5.77 | 3.56 | 2.07 |
|  |  |  | b | | 1.13 | | 1.96 | | 1.16 | | 13.52 | | 10.82 | | 29.22 | | 21.13 | | 2.72 | 0.73 | 12.11 | 2.33 | 11.35 | 5.20 | 3.40 | 2.13 |
|  | **6** | | 2.41 | | 1.04 | | 2.13 | | 1.15 | | 24.62 | | 19.20 | | 36.23 | | 26.14 | | 3.93 | 0.91 | 14.88 | 3.11 | 9.38 | 4.81 | 3.81 | 2.06 |
|  |  |  | 2.17 | | b | | 1.84 | | 1.06 | | 22.19 | | 20.14 | | 36.94 | | 28.04 | | 3.99 | 0.77 | 12.69 | 2.99 | 10.65 | 4.91 | 3.66 | 2.31 |
|  | **7** | | 2.05 | | 1.16 | | 2.22 | | 1.04 | | 26.50 | | 22.06 | | 35.97 | | b | | 2.93 | 0.86 | 21.30 | 2.56 | 10.34 | 4.99 | 3.79 | 2.51 |
|  |  |  | 2.10 | | 1.02 | | b | | b | | 26.56 | | 20.42 | | 36.98 | | b | | 3.26 | 0.80 | 15.66 | 2.40 | 10.50 | 4.23 | 3.57 | 2.24 |
| **MEAN** | | | 2.13 | | 1.09 | | 1.99 | | 1.10 | | 24.26 | | 18.55 | | 38.13 | | 29.03 | | 3.55 | 0.77 | 15.39 | 2.45 | 13.09 | 5.43 | 3.91 | 2.34 |
| **STANDARD DEVIATION** | | | 0.12 | | 0.07 | | 0.12 | | 0.06 | | 5.40 | | 3.69 | | 5.82 | | 4.68 | | 0.59 | 0.16 | 3.17 | 0.37 | 2.47 | 0.69 | 0.47 | 0.27 |
| **%CV** | | | 5.66 | | 6.63 | | 6.23 | | 5.31 | | 22.27 | | 19.91 | | 15.26 | | 16.12 | | 16.48 | 21.19 | 20.62 | 15.21 | 18.91 | 12.73 | 12.01 | 11.34 |
|  | ^a^ concentrations (ug/L) prior to correcting for dilution factor are listed for each of the 7 plates. Controls were run in duplicate.  ^b^ Insufficient sample. Blank cells were not used for %CV calculation. | | | | | | | | | | | | | | | | | | | | | | | | | |
|  | |  | |  | |  | |  | |  | |  | |  | |  | |  | | | | | | | | |

**Supplemental Table 2. Sample characteristics, significance tests and AUC values for AGR2, SYCN, REG1B, LOXL2 and CA19.9 analyzed in Sample Set B for comparisons of PDAC versus benign and PDAC versus other cancers.**

| **PDAC^b^ versus benign** | **Marker** | **Sample Set** | **Median Benign** | **Median PDAC** | **Median Ratio** | **Wilcoxon  p-value**^a^ | **AUC**^b^ | **Lower 95% CI**^c^ | **Upper 95% CI** |
| --- | --- | --- | --- | --- | --- | --- | --- | --- | --- |
|  | **SYCN** | B | 7.88 | 12.72 | 1.61 | 0.014 | 0.64 | 0.53 | 0.74 |
|  | **AGR2** | B | 88.8 | 173.90 | 1.96 | 2.11E-06 | 0.76 | 0.67 | 0.85 |
|  | **REG1B** | B | 9344 | 25380 | 2.72 | 0.0085 | 0.64 | 0.54 | 0.75 |
|  | **LOXL2** | B | 150.8 | 110.70 | 0.73 | 0.377 | 0.55 | 0.44 | 0.65 |
|  | **CA19.9** | B | 8.73 | 144.63 | 16.57 | 1.44E-11 | 0.87 | 0.81 | 0.93 |
| **PDAC versus other cancers** | **Marker** | **Sample Set** | **Median Other Cancer** | **Median PDAC** | **Median Ratio** | **Wilcoxon  p-value** | **AUC** | **Lower 95% CI** | **Upper 95% CI** |
|  | **SYCN** | B | 11.16 | 12.72 | 1.14 | 0.89 | 0.51 | 0.41 | 0.60 |
|  | **AGR2** | B | 74.57 | 173.90 | 2.33 | 4.54E-10 | 0.79^d^ | 0.72 | 0.86 |
|  | **REG1B** | B | 16903 | 25380 | 1.50 | 0.13 | 0.57 | 0.48 | 0.66 |
|  | **LOXL2** | B | 163.6 | 110.7 | 0.68 | 0.056 | 0.59 | 0.50 | 0.68 |
|  | **CA19.9** | B | 10.04 | 144.63 | 14.41 | 3.86E-11 | 0.81^d^ | 0.74 | 0.88 |

**^a^** The p-value was obtained through the Mann-Whitney-Wilcoxon test.
^b^AUC, area under the receiver operating characteristic curve; PDAC, pancreatic ductal adenocarcinoma (analogous to use of the term ‘pancreatic cancer’ elsewhere in this report)
^c^Confidence intervals for AUC were calculated using DeLong’s method. Sample sizes are provided in Table 1 and disease characteristics of the “benign” and “other cancer” groups are provided in the Materials and Methods portion of the text.
^d^No significant difference was found between AUC of AGR2 and CA19.9 in distinguishing PDAC from other cancers (p=0.69).

**Supplemental Table 3. Association of biomarkers with age and gender.**

| **Marker** | **Age correlation**^a^ | | **Gender association**^b^ | | |
| --- | --- | --- | --- | --- | --- |
|  | **Rho** | **p-value** | **Median males** | **Median females** | **p-value** |
| **SYCN** | -0.113 | 0.361 | 3.3 | 3.85 | 0.086 |
| **AGR2** | 0.00579 | 0.963 | 173.3 | 189.2 | 0.180 |
| **REG1B** | 0.188 | 0.127 | 4150 | 4468 | 0.151 |
| **LOXL2** | -0.0177 | 0.887 | 147.8 | 136.8 | 0.327 |
| **CA19.9** | -0.0386 | 0.756 | 6 | 6 | 0.827 |

^a^Spearman’s rank correlation coefficient was assessed to determine the association of marker levels with age of samples in the healthy group. No significant correlation was noted.
^b^The Wilcoxon test was used to assess marker correlation with age; no significant correlation was noted.

**Supplemental Table 4. Biomarker modeling in PDAC versus benign disease.**

| **Biomarker Combination**^a^ | **AUC**^b^ **of Combination** | **Lower 95% Confidence Interval^c^** | **Upper 95% Confidence Interval** | **p-value of AUC of panel compared to AUC of CA19.9** |
| --- | --- | --- | --- | --- |
| CA19.9+REG1B | 0.88 | 0.82 | 0.92 | 0.0008 |
| CA19.9 | 0.87 | 0.81 | 0.93 | 1.0000 |
| CA19.9+SYCN+REG1B | 0.87 | 0.82 | 0.92 | 0.0301 |
| CA19.9+AGR2+REG1B | 0.87 | 0.82 | 0.92 | 0.0043 |
| CA19.9+REG1B+LOXL2 | 0.86 | 0.81 | 0.91 | 0.0640 |
| CA19.9+SYCN+AGR2 | 0.86 | 0.80 | 0.91 | 0.1292 |
| CA19.9+SYCN | 0.86 | 0.81 | 0.91 | 0.1471 |
| CA19.9+SYCN+LOXL2 | 0.85 | 0.79 | 0.90 | 0.2781 |
| CA19.9+AGR2 | 0.82 | 0.76 | 0.88 | 0.9463 |
| CA19.9+AGR2+LOXL2 | 0.80 | 0.74 | 0.86 | 0.2825 |
| CA19.9+LOXL2 | 0.80 | 0.74 | 0.86 | 0.2375 |
| SYCN+REG1B | 0.78 | 0.72 | 0.84 | 0.3065 |
| SYCN+REG1B+LOXL2 | 0.78 | 0.71 | 0.84 | 0.2506 |
| SYCN+AGR2+REG1B | 0.77 | 0.71 | 0.83 | 0.2341 |
| REG1B+LOXL2 | 0.75 | 0.68 | 0.81 | 0.0814 |
| AGR2+REG1B+LOXL2 | 0.71 | 0.64 | 0.78 | 0.0099 |
| SYCN+AGR2 | 0.71 | 0.63 | 0.78 | 0.0106 |
| SYCN+AGR2+LOXL2 | 0.70 | 0.63 | 0.77 | 0.0085 |
| SYCN+LOXL2 | 0.70 | 0.62 | 0.77 | 0.0084 |
| AGR2+REG1B | 0.68 | 0.60 | 0.75 | 0.0018 |
| AGR2+LOXL2 | 0.58 | 0.50 | 0.66 | <0.001 |

^a^ Biomarker models for two and three marker combinations generated in PDAC versus disease-free controls of Sample Set B and presented in Table 3 were validated in PDAC (n=82) versus healthy benign disease samples (n=41) of Sample Set B and ordered from greatest to lowest AUC.
^b^ AUC, area under the receiver operating characteristic curve; PDAC, pancreatic ductal adenocarcinoma
^c^ Confidence intervals for AUC were calculated using DeLong’s method. P-values were calculated by taking 2000 stratified bootstrap samples.

**Supplemental Table 5. Biomarker modeling in PDAC versus other cancers.**

| **Biomarker Combination**^a^ | **AUC**^b^ **of Combination** | **Lower 95% Confidence Interval**^c^ | **Upper 95% Confidence Interval** | **p-value of AUC of panel compared to AUC of CA19.9** |
| --- | --- | --- | --- | --- |
| CA19.9+AGR2 | 0.82 | 0.75 | 0.89 | 0.92 |
| CA19.9 | 0.81 | 0.74 | 0.88 | 1.00 |
| CA19.9+AGR2+REG1B | 0.79 | 0.72 | 0.86 | 0.48 |
| CA19.9+REG1B | 0.79 | 0.71 | 0.86 | 0.44 |
| CA19.9+AGR2+LOXL2 | 0.79 | 0.71 | 0.86 | 0.44 |
| CA19.9+LOXL2 | 0.78 | 0.71 | 0.86 | 0.42 |
| CA19.9+SYCN | 0.77 | 0.69 | 0.85 | 0.28 |
| CA19.9+REG1B+LOXL2 | 0.77 | 0.69 | 0.85 | 0.28 |
| CA19.9+SYCN+AGR2 | 0.77 | 0.69 | 0.84 | 0.24 |
| CA19.9+SYCN+REG1B | 0.76 | 0.68 | 0.84 | 0.20 |
| CA19.9+SYCN+LOXL2 | 0.76 | 0.67 | 0.84 | 0.18 |
| AGR2+LOXL2 | 0.73 | 0.65 | 0.82 | 0.08 |
| AGR2+REG1B | 0.65 | 0.56 | 0.74 | <0.01 |
| AGR2+REG1B+LOXL2 | 0.61 | 0.52 | 0.70 | <0.01 |
| SYCN+AGR2+REG1B | 0.58 | 0.48 | 0.67 | <0.01 |
| SYCN+AGR2 | 0.57 | 0.48 | 0.67 | <0.01 |
| SYCN+AGR2+LOXL2 | 0.57 | 0.47 | 0.66 | <0.01 |
| REG1B+LOXL2 | 0.55 | 0.46 | 0.64 | <0.01 |
| SYCN+REG1B+LOXL2 | 0.55 | 0.46 | 0.64 | <0.01 |
| SYCN+REG1B | 0.55 | 0.45 | 0.64 | <0.01 |
| SYCN+LOXL2 | 0.52 | 0.42 | 0.61 | <0.01 |

^a^ Biomarker models for two and three marker combinations generated in PDAC versus disease-free controls of Sample Set B and presented in Table 3 were validated in PDAC (n=82) versus other cancer samples (n=70) of Sample Set B and ordered from greatest to lowest AUC.
^b^ AUC, area under the receiver operating characteristic curve; PDAC, pancreatic ductal adenocarcinoma
^c^ Confidence intervals for AUC were calculated using DeLong’s method. P-values were calculated by taking 2000 stratified bootstrap samples.

**Supplemental Table 6.** **Assessment of marker performance in 69 PDAC samples with CA19.9 levels within normal limits (<37 Units/mL).**

| **Sample Set**^a^ | **Candidate** | **Median Ratio** | **Wilcoxon P-value** | **AUC**^c^ **(95% Confidence Intervals)** | **Specificity at 90% sensitivity** | **Sensitivity at 90% Specificity** |
| --- | --- | --- | --- | --- | --- | --- |
| B | SYCN^b^ | 3.49 | <0.01 | 0.84 (0.74-0.93) | 0.65 | 0.38 |
|  | REG1B | 3.67 | <0.01 | 0.73 (0.60-0.86) | 0.25 | 0.39 |
|  | CA19.9 | 0.94 | 0.40 | 0.56 (0.42-0.71) | 0.14 | 0.21 |
|  | LOXL2 | 0.97 | 0.88 | 0.51 (0.36-0.67) | 0.01 | 0.04 |
|  | AGR2 | 1.06 | 0.81 | 0.52 (0.37-0.67) | 0.10 | 0.11 |
| A | SYCN^b^ | 2.09 | <0.01 | 0.67 (0.57-0.77) | 0.21 | 0.29 |
|  | REG1B | 2.26 | <0.01 | 0.66 (0.56-0.76) | 0.24 | 0.25 |
|  | CA19.9 | 1.33 | 0.02 | 0.61 (0.52-0.70) | 0.16 | 0.09 |
|  | LOXL2 | 1.12 | 0.14 | 0.58 (0.48-0.68) | 0.20 | 0.11 |
|  | AGR2 | 0.89 | 0.42 | 0.54 (0.44-0.65) | 0.05 | 0.09 |

^a^Of the total 432 samples, 182 were PDAC and of those, 69 (38%) had CA19.9 levels that were within normal limits (n=45 in Sample Set A and n=24 in Sample Set B). Comparisons between these samples and healthy controls of Sample Set A (n=92) and non-cancer controls of Sample Set B (n=47) are displayed, along with Wilcoxon p-values.
^b^SYCN showed the largest improvement in AUC in the set of PDAC samples with normal CA19.9 levels, with the ability to capture 13 and 8 PDAC cases missed by CA19.9 in Sample Sets A and B, respectively, at a specificity of 90%.
^c^ AUC, area under the curve from receiver operating characteristic analysis

**Supplemental Table 7. Marker performance in Early Stage (I/II) versus Healthy of Sample Set A.**

| **Marker/Combination**^a^ | **AUC**^b^ | **Lower Limit of 95% CI**^c^ | **Upper Limit of 95% CI** | **Specificity at 95% Sensitivity** | **Sensitivity at 95% Specificity** |
| --- | --- | --- | --- | --- | --- |
| CA19.9+SYCN+REG1B | 0.871 | 0.792 | 0.950 | 0.489 | 0.390 |
| CA19.9+SYCN+AGR2 | 0.849 | 0.747 | 0.951 | 0.310 | 0.500 |
| CA19.9+SYCN+LOXL2 | 0.846 | 0.757 | 0.934 | 0.467 | 0.400 |
| CA19.9+SYCN | 0.841 | 0.751 | 0.932 | 0.451 | 0.400 |
| CA19.9+AGR2+REG1B | 0.835 | 0.738 | 0.932 | 0.348 | 0.350 |
| SYCN+AGR2+REG1B | 0.822 | 0.725 | 0.919 | 0.375 | 0.400 |
| CA19.9+REG1B | 0.816 | 0.724 | 0.909 | 0.457 | 0.350 |
| CA19.9+REG1B+LOXL2 | 0.816 | 0.724 | 0.908 | 0.462 | 0.350 |
| CA19.9+AGR2 | 0.796 | 0.674 | 0.918 | 0.163 | 0.350 |
| CA19.9+AGR2+LOXL2 | 0.796 | 0.673 | 0.918 | 0.147 | 0.350 |
| SYCN+REG1B | 0.794 | 0.688 | 0.900 | 0.353 | 0.400 |
| SYCN+REG1B+LOXL2 | 0.793 | 0.683 | 0.903 | 0.293 | 0.415 |
| CA19.9+LOXL2 | 0.774 | 0.654 | 0.894 | 0.251 | 0.350 |
| CA19.9 | 0.758 | 0.634 | 0.881 | 0.134 | 0.350 |
| SYCN+AGR2+LOXL2 | 0.740 | 0.605 | 0.874 | 0.130 | 0.290 |
| SYCN+AGR2 | 0.738 | 0.603 | 0.872 | 0.120 | 0.250 |
| SYCN | 0.734 | 0.601 | 0.867 | 0.130 | 0.250 |
| SYCN+LOXL2 | 0.734 | 0.599 | 0.868 | 0.136 | 0.280 |
| AGR2+REG1B | 0.701 | 0.585 | 0.816 | 0.408 | 0.270 |
| AGR2+REG1B+LOXL2 | 0.701 | 0.585 | 0.816 | 0.408 | 0.270 |
| REG1B | 0.653 | 0.510 | 0.795 | 0.141 | 0.205 |
| REG1B+LOXL2 | 0.636 | 0.485 | 0.786 | 0.060 | 0.205 |
| AGR2 | 0.596 | 0.470 | 0.721 | 0.076 | 0.100 |
| AGR2+LOXL2 | 0.593 | 0.467 | 0.718 | 0.076 | 0.100 |
| LOXL2 | 0.486 | 0.342 | 0.631 | 0.054 | 0.000 |

^a^ All markers and combinations of two and three markers were assessed in PDAC samples with confirmed early stage (n=20) and healthy controls (n=92). Markers/combinations are ordered from greatest to least AUC.
^b^ AUC, area under the receiver operating characteristic curve; PDAC, pancreatic ductal adenocarcinoma
^c^ Confidence intervals (CI) for AUC were calculated using DeLong’s method.

**Supplemental Table 8. Marker performance in Early Stage (I/II) versus Disease-free of Sample Set B.**

| **Marker/Combination**^a^ | **AUC**^b^ | **Lower Limit of 95% CI**^c^ | **Upper Limit of 95% CI** | **Specificity at 95% Sensitivity** | **Sensitivity at 95% Specificity** |
| --- | --- | --- | --- | --- | --- |
| CA19.9+SYCN+REG1B | 0.917 | 0.860 | 0.974 | 0.489 | 0.683 |
| CA19.9+SYCN | 0.907 | 0.847 | 0.967 | 0.522 | 0.724 |
| CA19.9+SYCN+AGR2 | 0.905 | 0.844 | 0.966 | 0.500 | 0.724 |
| CA19.9+SYCN+LOXL2 | 0.905 | 0.844 | 0.967 | 0.489 | 0.733 |
| CA19.9+REG1B+LOXL2 | 0.857 | 0.767 | 0.947 | 0.149 | 0.689 |
| CA19.9+REG1B | 0.855 | 0.766 | 0.943 | 0.181 | 0.668 |
| CA19.9+AGR2+REG1B | 0.853 | 0.762 | 0.943 | 0.181 | 0.651 |
| SYCN+REG1B+LOXL2 | 0.843 | 0.761 | 0.925 | 0.413 | 0.429 |
| SYCN+REG1B | 0.841 | 0.758 | 0.924 | 0.402 | 0.404 |
| SYCN+AGR2+REG1B | 0.841 | 0.758 | 0.924 | 0.435 | 0.404 |
| SYCN+AGR2 | 0.818 | 0.728 | 0.908 | 0.359 | 0.324 |
| SYCN+AGR2+LOXL2 | 0.817 | 0.727 | 0.907 | 0.326 | 0.329 |
| SYCN+LOXL2 | 0.813 | 0.722 | 0.904 | 0.326 | 0.300 |
| SYCN | 0.810 | 0.717 | 0.902 | 0.326 | 0.250 |
| CA19.9+LOXL2 | 0.806 | 0.701 | 0.912 | 0.053 | 0.692 |
| CA19.9+AGR2 | 0.804 | 0.698 | 0.909 | 0.043 | 0.675 |
| CA19.9+AGR2+LOXL2 | 0.804 | 0.698 | 0.910 | 0.053 | 0.692 |
| CA19.9 | 0.803 | 0.699 | 0.907 | 0.043 | 0.667 |
| REG1B | 0.791 | 0.695 | 0.888 | 0.245 | 0.324 |
| REG1B+LOXL2 | 0.785 | 0.685 | 0.884 | 0.160 | 0.370 |
| AGR2+REG1B+LOXL2 | 0.782 | 0.685 | 0.879 | 0.309 | 0.340 |
| AGR2+REG1B | 0.780 | 0.682 | 0.877 | 0.298 | 0.318 |
| AGR2 | 0.644 | 0.525 | 0.764 | 0.085 | 0.200 |
| AGR2+LOXL2 | 0.637 | 0.516 | 0.757 | 0.096 | 0.200 |
| LOXL2 | 0.557 | 0.431 | 0.683 | 0.000 | 0.096 |

^a^ All markers and combinations of two and three markers were assessed in PDAC samples with confirmed early stage (n=40) and healthy controls (n=47). Markers/combinations are ordered from greatest to least AUC.
^b^ AUC, area under the receiver operating characteristic curve; PDAC, pancreatic ductal adenocarcinoma
^c^ Confidence intervals (CI) for AUC were calculated using DeLong’s method.
